# Mining expansin-like proteins from rumen microbiota and functional characterization of two anaerobic fungal expansin-like proteins

**DOI:** 10.1186/s40104-025-01287-6

**Published:** 2025-11-20

**Authors:** Hongjian Dai, Jian Gao, Yuling Wei, Qi Wang, Weiyun Zhu, Yanfen Cheng

**Affiliations:** 1 Laboratory of Gastrointestinal Microbiology, National Centre for International Research On Animal Gut Nutrition, Nanjing Agricultural University, Nanjing, 210095 China; 2 Center for Grassland Microbiome, College of Pastoral Agriculture Science and Technology, Lanzhou University, Lanzhou, 730000 China

**Keywords:** Anaerobic fungi, Expansin, Lignocellulose degradation, Multi-omics analysis

## Abstract

**Background:**

Sustainable livestock production is essential for food security and environmental management. Lignocellulosic biomass can be used in animal feed, thereby reducing feed production costs and enhancing sustainability. Expansin-like proteins (ELPs) play essential roles in plant cell wall degradation, yet their functions remain largely underexplored in rumen microbes. The purpose of this study was to investigate the effects of rumen microbial ELPs on lignocellulose degradation.

**Results:**

This study systematically identified 396 ELPs within the rumen microbiota, uncovering remarkable diversity, particularly among anaerobic fungi. Three representative ELPs from *Pecoramyces ruminantium* F1 (*PF*Loos_1, *PF*SWO1_1, *PF*SWO2_1) were selected for biochemical characterization. While *PF*SWO2_1 could not be expressed, *PF*Loos_1 and *PF*SWO1_1 exhibited significant synergy with cellulases. The CBM10-containing *PF*SWO1_1 demonstrated superior thermal stability (up to 65 °C) and substrate affinity, increasing rice straw hydrolysis efficiency by 21.6% (reducing sugar yield) compared to cellulase alone. Structural analyses revealed that CBM10 enabled *PF*SWO1_1 to preferentially bind complex substrates, whereas the single-domain *PF*Loos_1 targeted simpler substrates. Notably, ELP pretreatment of corn stover significantly improved fermentation quality (pH and lactic acid) and nutritional value (neutral detergent fiber, acid detergent fiber, and water-soluble carbohydrates).

**Conclusions:**

These findings indicate that ELPs are abundant in the rumen and play a synergistic role in lignocellulosic biomass conversion.

**Supplementary Information:**

The online version contains supplementary material available at 10.1186/s40104-025-01287-6.

## Background

Lignocellulose is the most abundant renewable carbon source on Earth, serving as a vital feedstock for sustainable development of animal husbandry [1]. However, its structural complexity—signified by highly ordered cellulose microfibrils (crystalline cellulose) embedded within a cross-linked lignin–hemicellulose matrix—poses significant challenges for ruminants to digest and utilize [2]. Enzymatic pretreatment has emerged as the most promising method to improve the utilization efficiency of lignocellulose, offering key advantages such as precise substrate specificity, mild reaction conditions, and environmental sustainability [3]. Despite these advantages, conventional enzyme systems perform suboptimally on native lignocellulosic substrates, largely due to their limited ability to disrupt crystalline cellulose domains and overcome steric hindrance imposed by lignin. Although optimized enzyme cocktails can partially alleviate these limitations, the high cost of enzymes remains a major barrier to large-scale livestock farming [4]. These challenges have spurred growing interest in accessory proteins, which improve substrate accessibility by loosening plant cell walls and disrupting lignin–cellulose associations. This increased accessibility enables cellulases to act more efficiently, thereby reducing the required enzyme dosage and partially substituting for costly hydrolytic enzymes, ultimately lowering the production cost of lignocellulose bioconversion [5].

Among the various accessory proteins, expansins and expansin-related proteins have gained attention for their ability to enhance lignocellulose degradation through non-hydrolytic disruption of cellulose microfibrils, thereby improving cellulase accessibility [6]. First discovered in plant systems, where they mediate acid-induced cell wall extension, these proteins have since been identified across diverse microbial taxa through advanced genomic analyses [7, 8]. Microbial expansin-like proteins (ELPs, such as EXLX, swollenin and loosenins) have demonstrated remarkable biophysical activity, inducing measurable swelling and structural destabilization in various cellulose substrates such as mercerized cotton, microcrystalline cellulose (Avicel PH-101), and Whatman filter paper [9–12]. Importantly, these proteins differ in domain composition, which underlies their functional diversity: plant expansins and microbial ELPs typically share a core two-domain structure consisting of an N-terminal D1 domain (a six-stranded double-psi β-barrel structurally homologous to GH45) and a C-terminal D2 β-sandwich domain. In contrast, loosenins and ceratoplatanins lack the D2 domain, retaining only D1, whereas swollenins include additional domains such as fibronectin III beyond D1 and D2. These structural variations are associated with differences in substrate specificity and mechanisms of cellulose disruption. The rumen is an important bioreactor for lignocellulose degradation, playing a key role in the efficient breakdown of complex biomass [13]. This efficiency is facilitated by a sophisticated enzymatic arsenal that synergistically combines core cellulolytic enzymes with various accessory proteins, including ferulic acid esterases (FAEs), lytic polysaccharide monooxygenases (LPMOs), and expansin-like proteins [13–15]. Although several ELPs from ruminal fungi have been characterized in previous studies [15], the diversity and functional potential of these proteins in the rumen microbiome remain largely unexplored.

To address this knowledge gap, we employed an integrated multi-omics approach combined with biochemical validation. By analyzing rumen metagenomic datasets and microbial isolate genomes, we identified rich diversity of expansin-like genes across multiple rumen microbial lineages. Metatranscriptomic profiling further revealed pronounced expression of these genes, particularly in rumen fungal populations. Using the model rumen fungus *Pecoramyces ruminantium* F1 [16], we demonstrated that expression of ELPs is strongly induced during growth on grass substrates. Subsequent heterologous expression and biochemical characterization confirmed that the identified proteins possess significant cellulose-disrupting activity, providing mechanistic insights into their role in lignocellulose degradation.

## Materials and methods

### Main strains and reagents

The anaerobic fungal strain *Pecoramyces ruminantium* F1, originally isolated from goat rumen [17] and maintained in our laboratory culture collection, was cultured in modified M2 medium supplemented with 1% (w/v) mixed sugars as described previously [18]. For heterologous protein expression, we used the pPICZα expression vector (Invitrogen), which was propagated in *Escherichia coli* DH5α competent cells (Sangon Biotech, Shanghai, China) following standard protocols. The methylotrophic yeast *Pichia pastoris* GS115 (maintained in our laboratory stock) was used to serve as the expression host. Purified xylanase (5.2 × 10^4^ U/g) and cellulase (5.8 × 10^4^ U/g) preparations were provided by Ms. Yuping Ma and Dr. Yuqi Li (Laboratory of Gastrointestinal Microbiology, Nanjing Agricultural University, Nanjing, China), respectively. The corresponding nucleotide and amino acid sequences of these enzymes have been characterized previously and deposited in our earlier publication [19].

### Establishment of rumen expansin-like proteins dataset

The complete amino acid sequences of microbial ELPs were systematically retrieved from the UniProt database (Release 2023_03, https://www.uniprot.org/). These reference sequences were used as queries for comprehensive BLASTP searches (E-value cutoff ≤ 1e-5) against the NCBI non-redundant (NR) protein database (Version 2024-02-07) [20]. Candidate sequences were rigorously validated as authentic ELPs through dual confirmation using both the Expansin Engineering Database (ExED) [21] and the Expansin Gene Family Database (ExGFD) [22]. In parallel, the sequences were subjected to domain annotation via CD-Search [23] which incorporates information from multiple domain databases, including Pfam and other resources covered by InterPro, ensuring comprehensive domain coverage. The final expansin-like proteins was obtained by combining sequences identified through both the ExED/ExGFD validation and CD-Search annotation. All putative expansin-like sequences were classified taxonomically using DIAMOND (v2.0.15) [24] with default parameters, and only sequences confirmed to be microbial origin were retained. The resulting curated collection was then used to investigate three complementary data resources: (1) 952 rumen metagenomes processed through our standardized pipeline [25], (2) 487 cultured rumen microbial genomes (410 bacterial, 12 anaerobic fungal, and 65 protozoan), and (3) 46,831 metagenome-assembled genomes (MAGs) from rumen ecosystems (see supplementary Table S2 for data resources). All newly identified rumen-derived sequences were subjected to the same validation protocol and clustered at 95% identity using CD-HIT [26] to eliminate redundancy. This systematic, multi-tiered approach yielded the specialized Rumen Expansin-like Protein Dataset (REPD), a specialized resource that integrates comprehensive sequence annotations with detailed phylogenetic classifications and structural domain information.

### Analysis of the expression of expansin-like genes in rumen metatranscriptome based on REPD

We analyzed 74 rumen metatranscriptomic datasets publicly available from the previous studies (Table S4) to investigate the expression profiles of expansin-like genes in rumen. Following our established bioinformatics pipeline [13], the raw sequence data was comprehensively processed including: (1) quality control using Trimmomatic (v0.39), (2) host DNA removal using Bowtie2, and (3) de novo assembly via MEGAHIT (v1.2.9). The assembled contigs were systematically annotated against our REPD using DIAMOND (v2.0.15) [24] in BLASTX mode with sensitive parameters (e-value ≤ 1 × 10^–5^, identity ≥ 30%, coverage ≥ 50%). For each putative expansin-like gene, only the top-scoring alignment (best hit) was retained to ensure accurate annotation. Gene expression levels were quantified based on normalized read counts (transcripts per million, TPM) [27] and subsequently processed using a custom Python (v3.8) script.

### Genome sequencing and assembly of the *Pecoramyces ruminantium* F1 strain

The anaerobic fungal strain *Pecoramyces ruminantium* F1 was cultured in modified M2 medium under strictly anaerobic conditions according to the established protocols [16]. Fungal biomass was harvested during late-log phase of growth for genomic DNA extraction using an optimized CTAB method [28], which incorporated mechanical cell disruption with liquid nitrogen followed by lysis in CTAB buffer containing β-mercaptoethanol. The extracted DNA was subjected to rigorous quality control, including agarose gel electrophoresis and fluorometric quantification, to ensure high molecular weight (> 20 kb) and purity (OD_260/280_ ratio 1.8–2.0) prior to library construction. High-quality DNA samples were used to prepare sequencing libraries following manufacturer’s protocols for both PacBio Sequel II (SMRTbell Express Template Prep Kit v2.0) and Nanopore GridION (Ligation Sequencing Kit SQK-LSK109) platforms. Sequencing was performed using PacBio CCS mode for high-fidelity reads and Nanopore R9.4.1 flow cells for long reads. The resulting sequencing data were processed through a hybrid assembly pipeline, starting with primary assemblies using Canu (v2.2) [29] and Flye (2.9.1) [30] with default parameters. This was followed by consensus generation using QuickMerge (v0.3) [31] and iterative polishing with Racon (v1.4.21; https://github.com/isovic/racon) and Pilon (v1.24) [32]. The final assembled genome was annotated using the MAKER2 pipeline [33], incorporating gene predictions from AUGUSTUS (v3.5.0) [34] and repeat masking with RepeatMasker [35] to generate a comprehensive genomic resource for this rumen fungal isolate. The data are available in PRJNA517297.

### RNA extraction and transcriptional analysis

The fermentation experiments were conducted following established protocols from our laboratory [13]. Briefly, cultures were grown in 180-mL serum bottles inoculated with 10 mL of 3-day-old monocultures into 90 mL sterile modified M2 medium containing either 1.0 g simple sugar mixture (19% arabinose + 31% xylose + 50% glucose; AXG) or rice straw (RS) or wheat straw (WS) as substrates, with four replicates per group. Growth was monitored using pressure transducers as previously described [36], with sampling performed at the terminal logarithmic phase (48 h) following established phase determination methods based on gas production curves. Subsequent processing such as centrifugation (10,000 × *g*, 4 °C, 8 min), PBS washing, and liquid nitrogen storage for RNA preservation was carried out as reported previously [37].

RNA extraction was performed using TRIzol reagent (Invitrogen), and library preparation for BGISEQ-500 sequencing was performed following the published RNA-Seq protocol [37]. Bioinformatics analysis, including read mapping with Bowtie2 [38], read counting with featureCounts [39], and differential expression analysis using DESeq2 [40] was conducted following our standard pipeline [13]. Differential expression screening of REP-encoding genes against the *Pecoramyces ruminantium* F1 genome annotation was performed following the methodology outlined in our previous work [13], focusing on genes showing both significant expression levels (counts) and up-regulation (Log_2_(fold change)) in complex substrate groups (WS and RS) compared to the AXG group.

### Heterologous expression and purification of fungal expansin-like proteins

Three expansin-like genes (*PF*Loos1, *PF*SWO1_1 and *PF*SWO2_1) were identified from *Pecoramyces ruminantium* F1 and codon-optimized for *Pichia pastoris* expression. The genes were cloned into pPICZαA vector and transformed into *E. coli* DH5α for amplification. Following sequence verification, SalI-linearized plasmids were electroporated into *P. pastoris* GS115. Transformants were selected on minimal dextrose (MD) solid medium and verified by PCR.

For protein production, positive clones were cultured in buffered glycerol-complex medium (BMGY) until OD_600_ reached 6–10. The cultures were then induced in BMMY medium by adding 1% methanol every 12 h for a total of 120 h. The supernatant was collected by centrifugation (12,000 × *g*) and purified using Ni–NTA affinity chromatography. Proteins were eluted with imidazole gradient (10–250 mmol/L) in Tris-HCl buffer (pH 8.0) and analyzed using SDS-PAGE. Physicochemical properties were predicted using ProtParam. The detailed protocols are available in our previous study [13, 19].

### Functional characterization of recombinant fungal expansin-like proteins

Filter paper samples were incubated with fungal ELPs (*PF*Loos1 and *PF*SWO1_1) at a final concentration of 10 μg/mL in 50 mmol/L citrate buffer (pH 5.0) at 39 °C for 1 h, without the addition of any enzymes. The treated samples were then subjected to critical point drying using liquid CO_2_, sputter-coated with 10 nm Au/Pd alloy, and imaged by field-emission scanning electron microscopy (FE-SEM; HITACHI SU8100) at 5 kV acceleration voltage and 5,000×  magnification to visualize protein-induced cellulose microfibril modifications [41]. To characterize the enzymatic enhancement potential of fungal ELPs, comprehensive hydrolysis assays was performed under physiologically relevant conditions (39 °C, pH 6.8). Triplicate 1.5 mL reaction systems, containing 50 mmol/L citrate buffer, 10 mg filter paper (Whatman No. 1), 7.57 mU/mL xylanase (EC 3.2.1.8), 2.37 mU/mL cellulase (EC 3.2.1.4), and 0.5 mg/mL target protein, were incubated for 60 min with agitation at 150 r/min [13]. Hydrolytic efficiency was determined by quantifying reducing sugar endpoints using the 3,5-dinitrosalicylic acid (DNS) method, with glucose serving as the standard. The reaction conditions were systematically evaluated by determining: (i) optimal temperature (30–60 °C), (ii) protein dosage (0.1–1.0 mg/g substrate), and (iii) pH (3.0–8.0) for maximal synergistic activity with cellulase [42]. Synergistic activity (%) = [(total reducing sugars released by both the enzyme and ELPs/reducing sugar released by the enzyme alone) − 1] × 100%. Two treatment regimens were compared: (1) simultaneous addition of enzymes and ELPs and (2) pre-treatment with ELPs followed by enzymatic hydrolysis [43]. The pretreatment was performed by incubating the ELPs with Whatman No. 1 filter paper at 55 °C and pH 5.0 for 1 h. Following this pretreatment, cellulase (or xylanase) was added to the reaction mixture, and the reaction was subsequently carried out at 39 °C for an additional 1 h. After completion, the amount of reducing sugars released was measured and calculated. Finally, filter paper strength was measured using a tensile tester to quantify structural modifications induced by ELPs treatment [44].

### Silage preparation and nutritional composition analysis

Corn straw was sourced from a farm in Nanjing, Jiangsu Province, China (119°58′21″ E, 32°25′37″ N, altitude 30.2 m). Prior to ensiling, the nutritional composition of the corn straw was determined: neutral detergent fiber (NDF, 69.64% ± 0.33%), acid detergent fiber (ADF, 47.96% ± 0.26%) and water-soluble carbohydrates (WSC, 12.27% ± 0.58%). The straw was chopped into 1–2 cm segments using a forage chopper. The treatments were as follows: (1) Corn straw + 2 × 10^3^ U cellulase + 1 × 10^6^ U xylanase (CON); (2) Corn straw pretreated with *PF*Loos at 400 µg/g substrate, followed by 2 × 10^3^ U cellulase + 1 × 10^6^ U xylanase (*PF*Loos_1); (3) Corn straw pretreated with *PF*SWO1_1 at 600 µg/g substrate, followed by 2 × 10^3^ U cellulase + 1 × 10^6^ U xylanase (*PF*SWO1_1). The dosage of cellulase and xylanase (1%) was selected based on a previous study [45]. Approximately 500 g of each prepared feed mixture was thoroughly mixed and packed into laboratory-scale polyethylene silage bags. The bags were vacuum-sealed using an extractor. A total of 12 bags were prepared (3 treatments × 4 replicates) and stored at ambient temperature (~ 30 °C).

Samples were collected after 30 days of ensiling. Fermentation parameters, including lactic acid and acetic acid, were analyzed using high-performance liquid chromatography (HPLC). The nutritional composition of the silage was assessed as follows: Neutral detergent fiber (NDF) and acid detergent fiber (ADF) contents were analyzed using the methods described by Van Soest et al. [46]. Water-soluble carbohydrate (WSC) content was measured according to a previously described method [47]. To prepare the extraction fluid for pH and ammonia-N determination, 10 g of fresh silage was homogenized with 90 mL of distilled water and stored at 4 °C for 24 h. The resulting filtrate was used to measure pH with a portable pH meter, and ammonia-N content was determined according to the method of Ke et al. [48].

### Statistical analysis

All quantitative data characterizing recombinant protein properties were subjected to one-way analysis of variance (ANOVA) using SPSS Statistics 22.0 (IBM, Armonk, NY, USA). Post-hoc comparisons of means were performed using Duncan’s multiple range test at a 95% confidence interval (*P* < 0.05). Results were expressed as mean ± standard error of the mean (SEM). Graphical representations were generated using GraphPad Prism 8.0 (GraphPad Software, San Diego, CA, USA), with statistical significance denoted by superscripted letters or asterisks, as appropriate. *P* < 0.05 was defined as the significance threshold.

## Results

### The ELP profiles of rumen microorganisms

Bioinformatics analysis revealed significant diversity in microbial expansin-like sequences. Initial sequence retrieval from UniProt yielded 64 fungal and 14 bacterial expansin-related sequences, which, after quality filtering, were refined to 46 fungal and 14 bacterial sequences. The NR database screening identified 4,863 microbial expansin-like sequences from 466,415,803, comprising 3,016 eukaryotic, 1,845 prokaryotic, and 2 archaeal representatives (Table S1). Phylogenetic analysis (Fig. S1) revealed clear evolutionary divergence between eukaryotic and prokaryotic ELPs, forming distinct phylogenetic clusters.

**Fig. 1:**
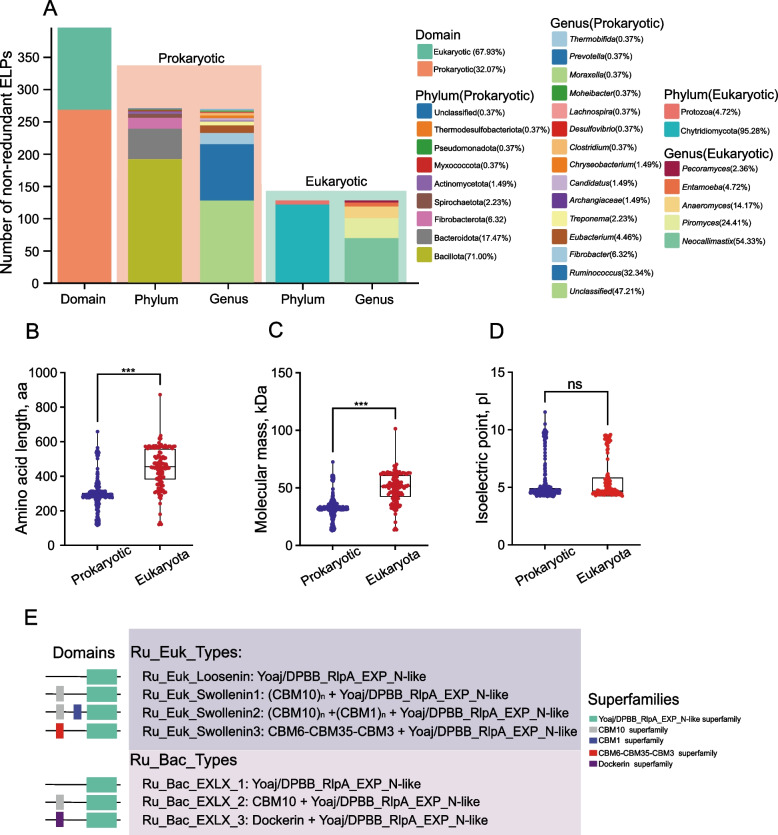
Characteristics of rumen microbial expansin-like proteins. **A** Taxonomic affiliations of identified ELPs of rumen microbial origin. Bars show the number of prokaryotic and eukaryotic sequences. **B–D** Comparative analysis between eukaryotic and prokaryotic ELPs: Sequence length (aa) (**B**), Molecular weight (kDa) (**C**) and Isoelectric point (pI) (**D**) distributions. ^***^P < 0.001. **E** Domain architecture classification of rumen ELPs. Eukaryotic and prokaryotic sequences are grouped by conserved domains (color-coded)

Through dereplication of 852 metagenome-derived and 553 genome-derived ELPs sequences, 396 non-redundant ELPs originating from rumen microbiota were identified. (Fig. S2A and B, Table S3). Taxonomic classification revealed that majority of sequences were of bacterial origin (269 sequences, 67.93%), primarily from Bacillota, while eukaryotic sequences (127, 32.07%) were predominantly from Chytridiomycota (Fig. 1A). At genus level, *Ruminococcus*, *Fibrobacter*, and unclassified rumen bacteria contributed the majority of bacterial ELPs, whereas *Neocallimastix*, *Piromyces*, and *Anaeromyces* were the major eukaryotic sources. Notably, 382 sequences (96.5%) showed < 95% amino acid identity with known NR database sequences, indicating substantial novelty in rumen microbial ELPs. Comparative analysis demonstrated significant differences between eukaryotic and bacterial ELPs. Eukaryotic variants possessed longer sequences and higher molecular weights (*P* < 0.05, Fig. 1B and C). The isoelectric points showed no significant variation between eukaryotic and bacterial ELPs (*P* > 0.05, Fig. 1D). Domain architecture analysis revealed conserved YoaJ and DPBB_RlpA_EXP_N superfamily domains across all sequences. Bacterial sequences uniquely contained Dockerin-like domains, while eukaryotic sequences featured carbohydrate-binding modules (CBMs). Based on domain organization, rumen microbial ELPs were classified into 7 distinct categories (4 eukaryotic and 3 prokaryotic; Fig. 1E).

### Expression profiles of ELPs in rumen metatranscriptomes

**Fig. 2:**
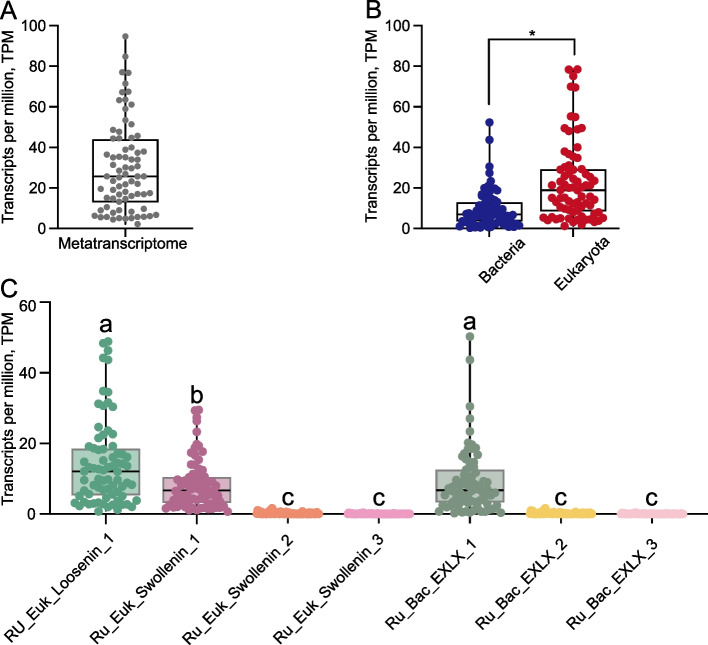
Metatranscriptomic profiling of expansin-like proteins in rumen microbiota. **A** Expression level of ELPs across rumen metatranscriptomes (*n* = 74). **B** Differential expression of ELPs derived from bacterial (blue) versus eukaryotic (red) microbial sources. ^*^P < 0.05. **C** Expression variation among ELP subtypes. ^a–c^Different lowercase letters indicate significant differences (*P* < 0.05). All expression values are normalized as transcripts per million (TPM)

Meta-omics analysis of rumen microbiota demonstrated distinct expression profiles of ELPs that are strongly associated with lignocellulose degradation potential. Quantification of ELP transcripts revealed a broad expression range (2.2–94.7 TPM) across different samples (Fig. 2A). Notably, ruminant species, such as buffalo, yak and roe deer, exhibiting superior roughage tolerance and lignocellulolytic activity demonstrated significantly elevated expression levels of ELP (Fig. S2B and C). Comparative analysis showed eukaryotic ELPs were expressed at substantially higher levels than their bacterial counterparts (*P* < 0.01, Fig. 2B). Among seven characterized ELP subtypes, RU_Euk_Loosenin and RU_Euk_Swollenin_1 emerged as the predominant eukaryotic forms, while RU_Bac_EXLX_1 was the major bacterial variant.

### Strain-resolved analysis of microbial ELPs in the rumen ecosystem

**Fig. 3:**
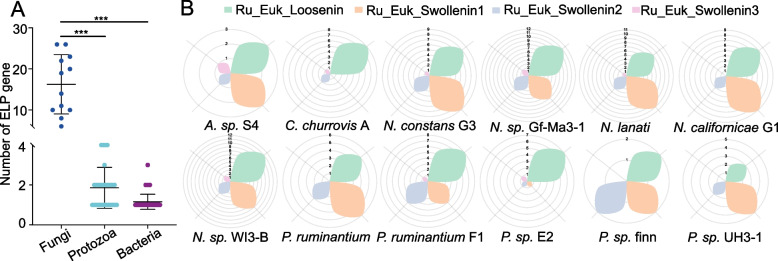
Distribution of expansin-like protein (ELP) encoding genes in rumen microbial strains. **A** Prevalence of ELP genes across major rumen microbial groups (*n* = 14,083 bacterial, 65 protozoal, and 12 anaerobic fungal genomes). ^***^P < 0.001. **B** Classification of ELP gene subtypes in anaerobic fungi (*n* = 12). *A. sp.* S4, *Anaeromyces sp.* S4; *C. churrovis* A, *Caecomyces churrovis* A; *N. constans* G3, *Neocallimastix constans* G3; *N. sp.* Gf-Ma3-1, *Neocallimastix sp.* Gf-Ma3-1; *N. lanate*, *Neocallimastix lanati*; *N. californicae* G1, *Neocallimastix californicae* G1; *N. sp.* WI3-B, *Neocallimastix sp.* WI3-B; *P. ruminantium*, *Pecoramyces ruminantium*; *P. ruminantium* F1, *Pecoramyces ruminantium* F1; *P. sp.* E2, *Piromyces sp.* E2; *P. sp.* Finn, *Piromyces sp.* finn; *P. sp.* UH3-1, *Piromyces sp*. UH3-1

To elucidate the genetic determinants underlying differential ELP expression patterns, a comprehensive survey of ELP-encoding genes across rumen microbial genomes was conducted. Bacterial genomes showed limited ELP carriage (3.8% prevalence; 1.16 genes/genome), contrasting sharply with anaerobic fungi (100% prevalence; 16.3 genes/genome) and protozoa (44.6% prevalence; 1.8 genes/genome) (Fig. 3A and Fig. S2A). Notably, the anaerobic fungal genus *Neocallimastix* exhibited exceptional ELP expansion (23.4 ± 3.2 genes/genome), surpassing protozoan (*Diplodinium*: 4.0 ± 1.1 genes) and bacterial (*Fibrobacter*: 1.3 ± 0.3 genes) counterparts by 5.8- and 18-fold, respectively (Fig. S4). Classification of 195 ELP genes from 12 anaerobic fungal genomes revealed that they predominantly belong to two evolutionarily distinct clades: RU_Euk_Loosenin (42.05%) and RU_Euk_Swollenin_1 (34.36%) (Fig. 3B). Species annotation results showed that protozoan ELPs exhibit mosaic origins, with *Epidinium_cattanei* SAG2 acquiring fungal-derived genes, while *Eremoplastron_rostratum* SAG1 containing bacterial-like variants-consistent with their dual ecological roles as both fiber degraders and bacterivores (Fig. S3). In contrast, bacterial and fungal ELPs showed vertical inheritance patterns congruent with their specialized lignocellulose-degrading niches.

### Substrate-induced expression of fungal ELPs in lignocellulose deconstruction

Our findings demonstrate a distinct transcriptional advantage of ELPs in rumen anaerobic fungi. Using the established model strain *Pecoramyces ruminantium* F1, technical challenges associated with its AT-rich genome (83.79% AT content) were overcome through optimized DNA extraction protocols and third-generation sequencing. The resulting high-quality genome assembly (29 contigs; largest contig 16.85 Mb; N50 8.73 Mb; Table S5) represents the most complete anaerobic fungal genome to date, encoding 21,577 genes including 42 ELP-coding sequences—the highest count observed among rumen microbial genomes (Compared to the results in Fig. S4).

**Fig. 4:**
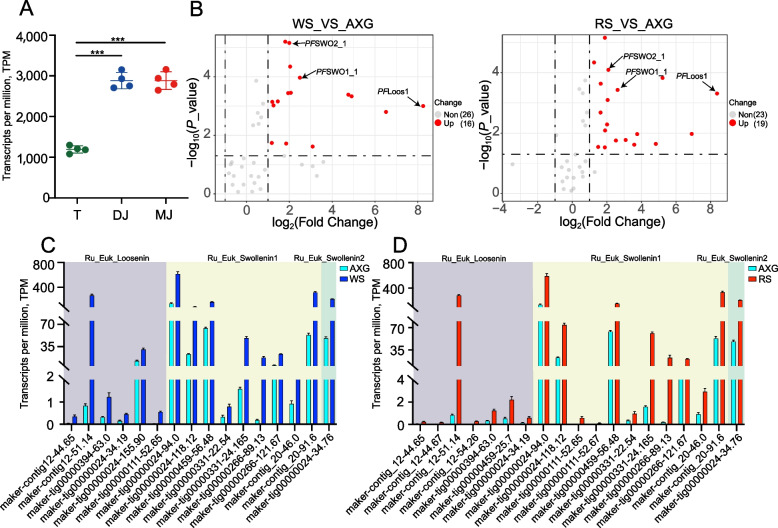
Identification and expression profiling of expansin-like protein (ELP) genes in *Pecoramyces ruminantium* F1. **A** Bar plot showing relative expression levels of ELP genes under different substrates: AXG: glucose + xylose + arabinose. RS: rice straw. WS: wheat straw. ^***^P < 0.001. **B** Volcano plot analysis of differentially expressed ELP genes (|log_2_FC| > 2, FDR < 0.05) across substrate conditions. Red dots indicate significantly upregulated genes. Classification and expression levels of differentially expressed ELP genes in wheat straw (**C**) and rice straw (**D**) substrates

Transcriptome analysis demonstrated distinct expression profiles of ELP genes in response to substrate complexity. Compared to monosaccharide cultures, growth on lignocellulosic substrates induced significant upregulation of 16 and 19 ELP genes (log_2_FC > 2, FDR < 0.05) during the degradation of wheat straw and rice straw, respectively (Fig. 4A and B). Type classification revealed differential representation among ELP subtypes: wheat straw preferentially induced RU_Euk_Loosenin isoforms (9 of 16 upregulated genes, 56.3%), while rice straw strongly activated both RU_Euk_Swollenin_1 (11 of 19 genes, 57.9%) and RU_Euk_Loosenin (7 of 19 genes, 36.8%) subtypes, with a single RU_Euk_Swollenin_2 gene also responding to rice straw (Fig. 4C and D). This substrate-dependent expression divergence suggests functional specialization within the ELP family, tailored todistinct plant cell wall architectures. Based on expression magnitude and ELP subtypes, three representative candidates for heterologous expression: *PF*Loos_1 (maker-contig12-51.14), *PF*SWO1_1 (maker-tig00000024-94.0), and *PF*SWO2_1 (maker-tig00000024-34.76) were selected (Fig. 4C and D).

### Heterologous expression and purification of ELPs from *Pecoramyces ruminantium* F1

**Fig. 5:**
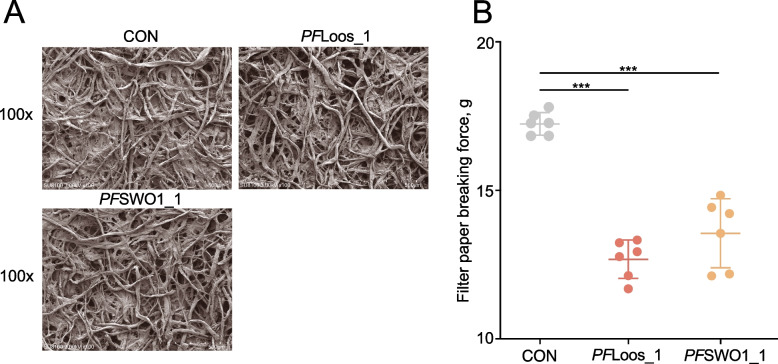
Weakening effect of expansin-like proteins on filter paper in *Pecoramyces ruminantium* F1. **A** Scanning electron micrographs showing microstructural alterations in filter paper following treatment with recombinant ELPs. Scale bar: 500 μm. **B** Quantitative analysis of tensile strength reduction in ELP-treated filter paper compared to controls. ^***^P < 0.001

BLASTP domain architecture analysis revealed distinct structural organizations among the three candidate proteins. *PF*Loos_1 contained a single YoaJ domain (belongs to RU_Euk_Loosenin), *PF*SWO1_1 possessed both CBM_10 and YoaJ domains (belongs to RU_Euk_Swollenin1), *PF*SWO2_1 featured a unique tripartite structure comprising CBM_10, CBM_1, and DPBB_RlpA_EXP_N-like domains (belonging to RU_Euk_Swollenin2, Fig. S5A). All three genes were successfully cloned into the pPICZα expression vector and transformed into *Pichia pastoris* GS115 competent cells. Following induction, two recombinant proteins (*PF*Loos_1 and *PF*SWO1_1) were expressed at levels sufficient for purification (> 40 mg/L culture) and subsequent characterization (Fig. S5B). However, *PF*SWO2_1 expression remained below detectable limits despite optimization attempts, suggesting potential translational or folding challenges associated with its complex domain architecture. Current methodologies for assessing microbial expansin activity primarily rely on non-enzymatic physical disruption assays, as these proteins operate through mechanical rather than hydrolytic mechanisms. Our results demonstrate that treatment with recombinant ELPs (*PF*Loos_1 and *PF*SWO1_1) significantly altered filter paper microstructure. Scanning electron microscopy revealed pronounced enlargement of interfibrillar spaces following protein incubation (Fig. 5A), while mechanical testing showed a significant reduction in tensile strength (*P* < 0.05, Fig. 5B).

### Biochemical characterization of *Pecoramyces ruminantium* F1 ELPs

**Fig. 6:**
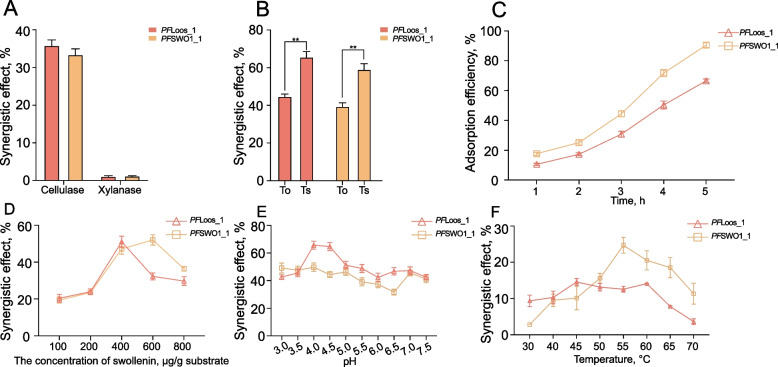
Biochemical characterization of expansin-like proteins from *Pecoramyces ruminantium* F1. **A** Synergistic enhancement of cellulase and xylanase activities by *PF*Loos_1 and *PF*SWO1_1. **B** Comparative analysis of synergistic efficiency under different protein addition strategies.^**^P < 0.01. To, simultaneous addition of enzymes and ELPs; Ts, pre-treatment with ELPs followed by enzymatic hydrolysis. **C** Substrate adsorption capacities of *PF*Loos_1 and *PF*SWO1_1. The optimal protein dosage (μg/g substrate) (**D**), pH (**E**) and temperature (**F**) of each ELP

In this experiment, using filter paper and xylan as model substrates, enhancement effects of *Pecoramyces ruminantium* F1-derived ELPs on cellulase and xylanase activities were analyzed. The recombinant proteins *PF*Loos_1 and *PF*SWO1_1 significantly enhanced cellulase efficiency, with activity increases of 35.5% ± 1.8% and 32.2% ± 1.6% respectively, after 1-h incubation (*P* < 0.05, Fig. 6A). In contrast, the enhancement of xylanase activity was minimal, with increases of only 1.3% ± 0.4% and 1.5% ± 0.2%. Pre-treatment of filter paper with ELPs (*PF*Loos_1 and *PF*SWO1_1) prior to cellulase addition demonstrated significantly higher catalytic efficiency compared to simultaneous application (*P* < 0.05, Fig. 6B). In addition, a comparative analysis of the substrate adsorption capacities between *PF*Loos_1 and *PF*SWO1_1 was also conducted. The results demonstrated a clear hierarchy in adsorption efficiency: *PF*SWO1_1 exhibited the higher substrate-binding affinity than *PF*Loos_1 (Fig. 6C). The optimal conditions for *PF*SWO1_1 was identified as 600 μg/g substrate, a temperature of 55 °C, and a pH of 4. In contrast, *PF*Loos_1 exhibited maximal activity at a lower dosage (400 μg/g substrate) and temperature (45 °C), while sharing the same optimal pH of 4.0 (Fig. 6 C–E).

### Effects of ELPs pretreatment on fermentation characteristics and nutrient composition of corn straw silage

Table 1 presents changes in pH, organic acid content, and NH_3_-N concentration of the silage. Compared to the CON group, all ELP-pretreated groups (*PF*Loos_1 and *PF*SWO1_1) exhibited significantly lower pH values (*P* < 0.05). Similarly, lactic acid content was significantly higher in both *PF*Loos_1 and *PF*SWO1_1 groups relative to the CON group (*P* < 0.05). Furthermore, ELP pretreatment significantly reduced NDF, ADF, WSC, and NH₃-N concentrations compared to the CON group (*P* < 0.05).

**Table 1:** Effects of ELP pretreatment on fermentation characteristics and nutrient composition of corn straw silage

Items	CON	*PF*Loos_1	*PF*SWO1_1	*P*-values
pH	3.73 ± 0.06^a^	3.52 ± 0.03^b^	3.46 ± 0.05^b^	< 0.001
Lactic acid, % DM	5.27 ± 1.06^a^	7.32 ± 0.96^b^	7.83 ± 1.21^b^	0.003
Acetic acid, % DM	1.30 ± 0.16	1.29 ± 0.23	1.32 ± 0.18	0.972
NDF, % DM	46.57 ± 1.56^a^	40.26 ± 1.79^b^	39.37 ± 1.45^b^	< 0.001
ADF, % DM	27.36 ± 1.62^a^	23.77 ± 0.92^b^	22.86 ± 1.77^b^	< 0.001
WSC, % DM	9.87 ± 1.07^a^	7.36 ± 0.83^b^	6.57 ± 1.12^b^	< 0.001
NH_3_-N, % DM	0.88 ± 0.02^a^	0.61 ± 0.01^b^	0.58 ± 0.03^b^	< 0.001

^a,b^Values within a row with different superscript letters differ significantly (*P* < 0.05). CON: Corn straw + 1% cellulase + 1% xylanase; *PF*Loos1: Corn straw pretreated with *PF*Loos + 1% cellulase + 1% xylanase; *PF*SWO1_1: Corn straw pretreated with *PF*SWO1_1 + 1% cellulase + 1% xylanase. *NDF* Neutral detergent fiber, *ADF* Acid detergent fiber, *WSC* Water-soluble carbohydrate. Data are presented as means ± SEM (*n* = 4)

## Discussion

The rumen plays a pivotal role in the efficient degradation of complex biomass and has long been recognized as a rich reservoir of hydrolytic enzymes [13]. However, with advances in sequencing and bioinformatics technologies, it has become increasingly evident that the rumen also harbors highly effective non-hydrolytic enzymes, such as pectate lyases [49], polygalacturonate lyases [50] and ELPs [15]. This highlights that the rumen is not only a source of classical hydrolases but also an untapped repertoire of novel enzyme families with distinct mechanisms of action, capable of facilitating plant cell wall deconstruction beyond conventional hydrolytic pathways. Despite this potential, more than 80% of rumen microorganisms remain uncultivable, making it challenging to comprehensively explore their genetic resources using traditional culture-based approaches. Moreover, the absence of a systematic dataset specifically dedicated to rumen microorganism derived ELPs has hindered further functional and mechanistic studies in this area. To overcome these limitations, we first compiled all known microbial ELP sequences into a curated reference gene set. Using this reference, we then systematically mined genome, metagenome, and MAGs data to identify ELPs from rumen microorganisms. This approach minimizes the loss of sequences during data processing and ensures the most comprehensive possible representation of rumen derived ELPs. Importantly, our strategy offers several advantages over conventional methods. By integrating multiple data sources, it enables the capture of highly divergent and low similarity ELP sequences that would otherwise be missed. It also expands the focus beyond hydrolytic CAZymes, highlighting the potential of non-hydrolytic proteins to enhance lignocellulose accessibility. Collectively, these efforts establish the first systematic rumen ELP dataset, providing a valuable resource for exploring novel enzyme families and advancing our understanding of non-hydrolytic mechanisms in biomass deconstruction.

This study reveals that rumen microorganisms involved in lignocellulose degradation (e.g., *Fibrobacter* and *Neocallimastix*) are enriched with ELPs encoding genes, indicating ELPs play a pivotal role in lignocellulose deconstruction. ELPs function by loosening the lignocellulosic structure, facilitating enhanced penetration and degradation by hydrolytic enzymes (cellulases and hemicellulases) [51]. Notably, ELPs from rumen microorganisms exhibit significantly lower average amino acid sequence identity to counterparts in public databases (UniProt and NR). This pronounced sequence divergence likely reflects evolutionary adaptations to the rumen environment. While anaerobic fungal genomes typically display elevated AT content, which may increase insertion/deletion mutation rates and partly explain the unique sequence features observed in fungal ELPs [52, 53]. The sequence divergence of bacterial ELPs is likely not driven by AT content. Instead, it appears to result from complex ecological selection pressures, such as adaptation to substrate-binding surfaces in high-fiber environments, and frequent horizontal gene transfer, leading to pronounced differences between rumen bacterial ELPs and those from other environments [20, 54]. This divergence is further evidenced by adaptive shifts in pI—alkaline ELPs predominate in Gram-positive bacteria, while acidic variants are more common in Gram-negative bacteria [54].

ELP gene expression strongly correlates with lignocellulose degradation potential. Ruminants exhibiting high roughage tolerance and lignocellulolytic activity, such as buffalo, yak, and roe deer, demonstrate significantly elevated ELP expression levels. This suggests ELP abundance may constitute a microbial adaptation mechanism to high-fiber environments, enhancing fiber degradation efficiency and thereby improving host nutrient acquisition from fibrous feeds [55]. Crucially, eukaryotic microbial ELPs (particularly from anaerobic fungi) showed significantly higher expression levels than bacterial ELPs, implying their dominant role in the initial stages of fiber degradation. This functional partitioning reflects fundamental niche differentiation: although bacteria constitute approximately 90% of rumen microbial biomass and dominate polysaccharide metabolism, anaerobic fungi possess unique mechanical penetration capabilities via rhizoidal growth [56]. This enables them to physically disrupt intact plant tissues and access microenvironments inaccessible to bacteria, accounting for their disproportionate contribution (~ 60%) to lignocellulose degradation [57, 58], likely mediated by these highly expressed ELPs. Moreover, ELP genes within rumen protozoa appear partially derived from bacteria and fungi, potentially acquired via horizontal gene transfer. Protozoal phagocytosis of microbes not only contributes to rumen ecosystem stability but may also facilitate the acquisition of degradative enzymes and ELP genes, enhancing overall organic matter degradation capacity [59].

Filter paper weakening capacity is a key metric for evaluating ELP activity. Our results confirm that ELPs significantly reduce filter paper breaking strength by disrupting the hydrogen-bonding network within cellulose [9, 42]. Mirroring findings for *Trichoderma harzianum* expansin *Tg*SWO [42, 43], our observation that ELPs exhibited stronger synergy with cellulases than with xylanases can be explained by their substrate preference. Previous studies demonstrated that ELPs, such as *Ccl*EXL1, bind more strongly to microcrystalline cellulose than to phosphoric acid-swollen cellulose (PASC), indicating a preferential interaction with crystalline cellulose regions rather than amorphous substrates [60]. Such preferential binding may explain why ELPs are particularly effective in enhancing cellulase activity on crystalline cellulose, whereas their impact on xylanases is less pronounced, given that hemicellulose is more amorphous and structurally accessible. The mode of addition also impacted synergy: a two-step process (ELP pretreatment followed by enzyme addition, Ts) outperformed simultaneous addition (To). Pretreatment likely allows ELPs to disrupt the substrate structure, increasing cellulose enzyme accessibility. In contrast, simultaneous addition may lead to competition for substrate binding sites and this interference could hinder cellulases from efficiently accessing the substrate, thereby diminishing the overall hydrolytic performance [61]. The presence of CBMs in ELPs generally enhances their substrate affinity by providing specific recognition and binding to polysaccharide surfaces. Accordingly, ELPs with CBM10 showed stronger adsorption to lignocellulosic substrates in our assays, whereas those lacking CBM10 exhibited comparatively lower binding. However, previous studies have demonstrated that CBM-independent ELPs can still associate with cellulose through alternative mechanisms, including entropy-driven processes [20, 62] as well as hydrophobic and electrostatic interactions [54]. Beyond substrate targeting, CBMs have also been reported to contribute to protein stability, such as improving tolerance to pH fluctuations and thermal stress [63, 64]. Such stabilizing effects may further support the functionality of CBM-containing ELPs during ruminal digestion, where enzymes are often exposed to variable environments. Consistent with these biochemical observations, our transcriptomic analysis revealed that in complex lignocellulosic substrates (WS and RS), the differentially expressed CAZymes were mainly associated with cellulose metabolism, including endoglucanases, exoglucanases, and β-glucosidases, with endoglucanases showing the most prominent changes. Since ELPs do not directly hydrolyze polysaccharides but loosen plant cell wall structures, their expression correlates functionally with the upregulation of these hydrolytic CAZymes, indicating a synergistic division of labor. This pattern also reinforces that ELPs preferentially act on crystalline cellulose, complementing the enzymatic action of cellulases. Furthermore, an optimal ELP dosage threshold exists; excessive amounts impede cellulose enzyme–substrate binding, reducing degradation efficiency [42].

The ability of ELPs to loosen lignocellulosic structures without directly hydrolyzing polysaccharides is particularly relevant for silage, where enhanced substrate accessibility strongly influences fermentation dynamics and forage quality. Organic acids, pH and NH₃-N are critical indicators of silage quality. In this study, ELP pretreatment significantly reduced NH₃-N content, indicating reduced proteolysis and nitrogen loss. Mechanistically, ELP-mediated degradation of plant cell wall cellulose releases fermentable sugars (glucose/disaccharides), promoting lactic acid bacteria proliferation [45]. Accelerated lactic acid accumulation lowers pH, inhibiting proteolytic activity by undesirable microbes and consequently reducing NH₃-N formation [65]. This explains the concomitant decrease in pH and increase in lactic acid observed in ELP-pretreated groups. Acetic acid and lactic acid enhance silage aerobic stability [66]. Increased lactic acid content in ELP-pretreated groups suggests benefits not only for ruminal degradability and energy conversion but also for improved feedstock stability. The changes of nutritional composition (e.g., NDF, ADF and WSC), collectively indicate enhanced feed nutritional value [67]. ELP pretreatment effectively disrupted the compact lignocellulosic structure, thereby providing ruminants with more digestible, high-nutrition forage. In this study, *PF*Loos_1, which only contains a single YoaJ domain and lacks CBM10, displayed comparable performance to *PF*SWO1_1 on both simple (filter paper) and complex (silage) substrates. This observation suggests that the absence of CBMs does not necessarily impair the cellulose-disrupting activity of ELPs [42]. One possible explanation is that the YoaJ domain itself possesses intrinsic affinity for cellulose, enabling *PF*Loos_1 to bind and loosen the cell wall matrix independently of CBM assistance [54, 62]. Moreover, ELPs are naturally acidic proteins that exhibit enhanced stability and activity under low-pH conditions [51]. Thus, the acidic environment of silage fermentation may provide particularly favorable conditions for *PF*Loos_1 function, helping to explain why its performance on silage was comparable to that of CBM ELPs. These findings demonstrate that the application of ELPs in livestock feed has practical significance: by increasing the digestibility of fibrous substrates, ELP pretreatment can reduce feed requirements, improve nutrient utilization, and thereby lower feeding costs. At the same time, more efficient utilization of agricultural residues contributes to sustainable livestock production by minimizing waste and enhancing energy conversion from plant biomass.

## Conclusion

This study established the first Rumen Expansin-like Protein Dataset (REPD), revealing a surprisingly diverse repertoire of ELPs in rumen microorganisms, particularly anaerobic fungi. Phylogenetic analysis suggests that horizontal gene transfer contributed to their distribution. Biochemical characterization of two representatives, *PF*Loos_1 and *PF*SWO1_1, demonstrated distinct properties: while CBM containing *PF*SWO1_1 showed strong substrate binding and stability, *PF*Loos_1, despite lacking CBM10, performed comparably across both simple and complex substrates, highlighting alternative structure function strategies. Collectively, these findings expand our understanding of rumen derived ELPs and provide a foundation for their potential application in improving fiber digestibility and promoting sustainable livestock production.

## Supplementary Information

Additional file 1: Table S1. Biochemical properties and taxonomic annotation of ELPs identified in the NR database. Table S2. Data sources of rumen microbial MAGs, isolate genomes, and metagenomes. Table S3. Biochemical properties, taxonomic annotation, and domain architecture of ELPs identified in rumen microorganisms. Table S4. Data sources of rumen metatranscriptomes used for ELPs analysis. Table S5. Genomic features of anaerobic fungi and *Pecoramyces ruminantium* F1. Supplemental fasta: The sequence of rumen expansin-like proteins.

Additional file 2: Fig. S1. Phylogenetic analysis of expansin-like proteins. Fig. S2. Identification and distribution of expansin-like proteinin rumen microbiomes. Fig. S3. Taxonomic origins of expansin-like proteinidentified from rumen microbial genomes. Fig. S4. Analysis of mean ELP-encoding gene counts per genome at the genus level for rumen bacteria, fungi, and protozoa. Fig. S5. Expression and purification of selected expansin-like proteinfrom *Pecoramyces ruminantium* F1.

## Data Availability

The genome data of *Pecoramyces ruminantium* F1 are available in PRJNA517297.

## Abbreviations

ADFD: Acid detergent fiber digestibility
CBMs: Carbohydrate-binding modules
CD: Cellulose digestibility
DMD: Dry matter digestibility
ELP: Expansin-like protein
HD: Hemicellulose digestibility
NDFD: Neutral detergent fiber digestibility
