# Supplementary material for: Mining expansin-like proteins from rumen microbiota and functional characterization of two anaerobic fungal expansin-like proteins
Source: J Anim Sci Biotechnol. 2025 Nov 20;16:155. doi: 10.1186/s40104-025-01287-6 (PMC12632131; doi:10.1186/s40104-025-01287-6)
Supplement: Supplementary file 2 — Additional file 2: Fig. S1. Phylogenetic analysis of expansin-like proteins. Fig. S2. Identification and distribution of expansin-like proteinin rumen microbiomes. Fig. S3. Taxonomic origins of expansin-like proteinidentified from rumen microbial genomes. Fig. S4. Analysis of mean ELP-encoding gene counts per genome at the genus level for rumen bacteria, fungi, and protozoa. Fig. S5. Expression and purification of selected expansin-like proteinfrom Pecoramyces ruminantium F1. [file 40104_2025_1287_MOESM2_ESM.docx]

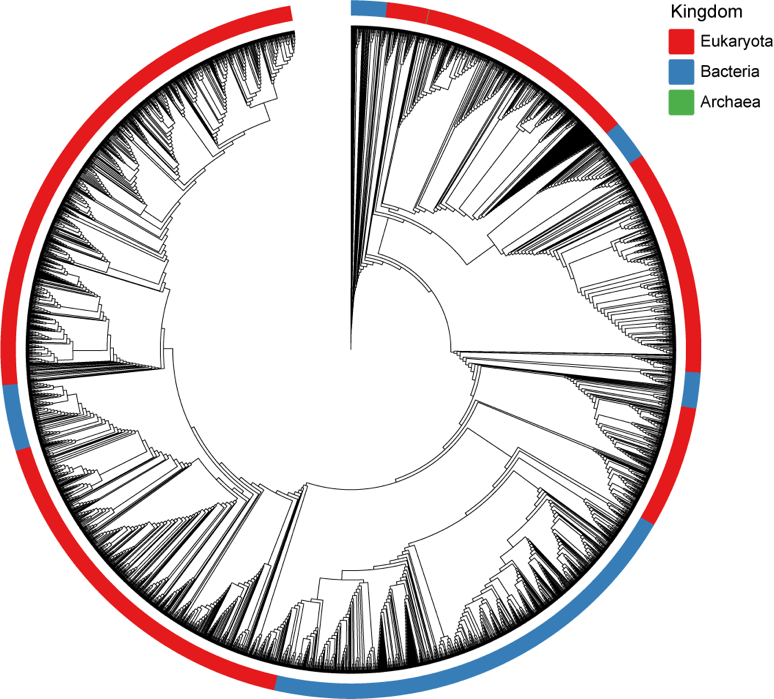


Figure S1. Phylogenetic analysis of expansin-like proteins. Maximum-likelihood tree showing evolutionary relationships of ELPs identified in the NCBI NR database. Proteins are color-coded by origin: eukaryotic microorganisms (red), bacteria (blue), and archaea (green).


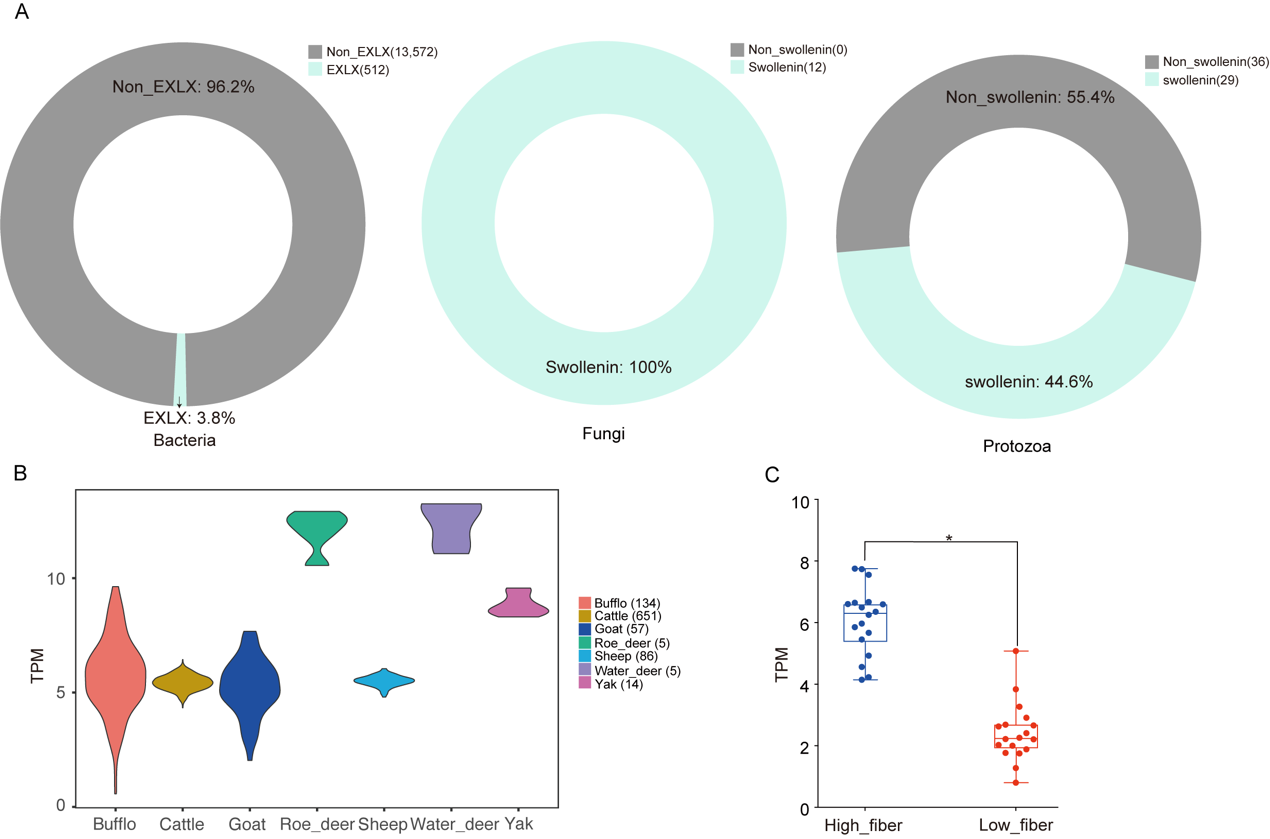


Figure S2. Identification and distribution of expansin-like protein (ELP) in rumen microbiomes. (A) Prevalence of ELP-coding genes across sequenced rumen microbial genomes, shown as percentage of genomes containing ELPs in bacterial, fungal and protozoal groups. (B) Relative abundance of ELP genes in rumen metagenomes from different ruminant species. (C) Differential expression of ELP genes correlated with dietary roughage ratio. Data derived from PRJEB23561 and PRJNA955930 repositories.


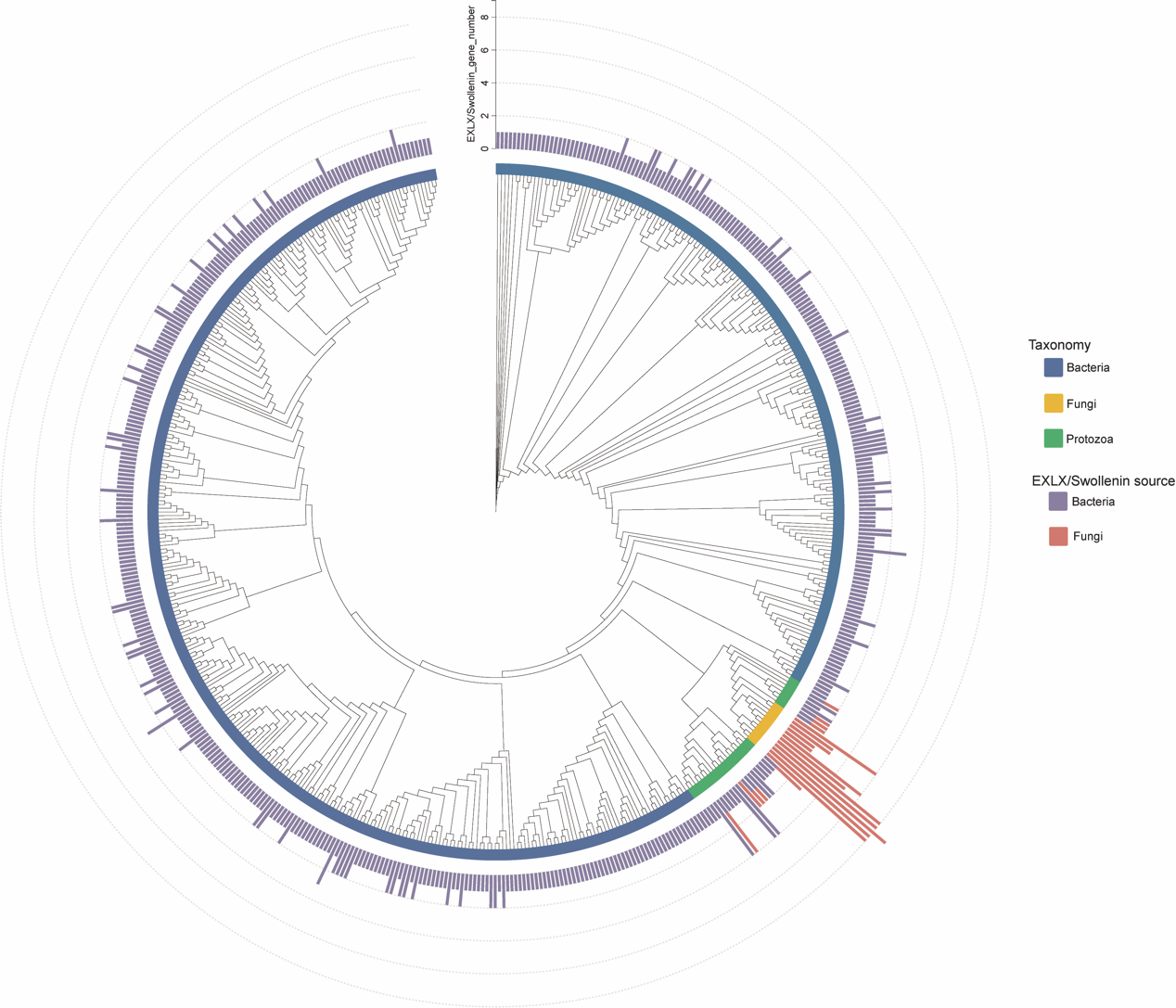


Figure S3. Taxonomic origins of expansin-like protein (ELP) identified from rumen microbial genomes. Phylogenetic tree constructed using 16S (bacterial) and 18S (fungal/protozoal) rRNA sequences (Circle graph: bacteria (blue), fungi (yellow), protozoa (green)), with ELP sequences overlaid (Bar graph: bacterial-derived (purple), fungal-derived(red)).


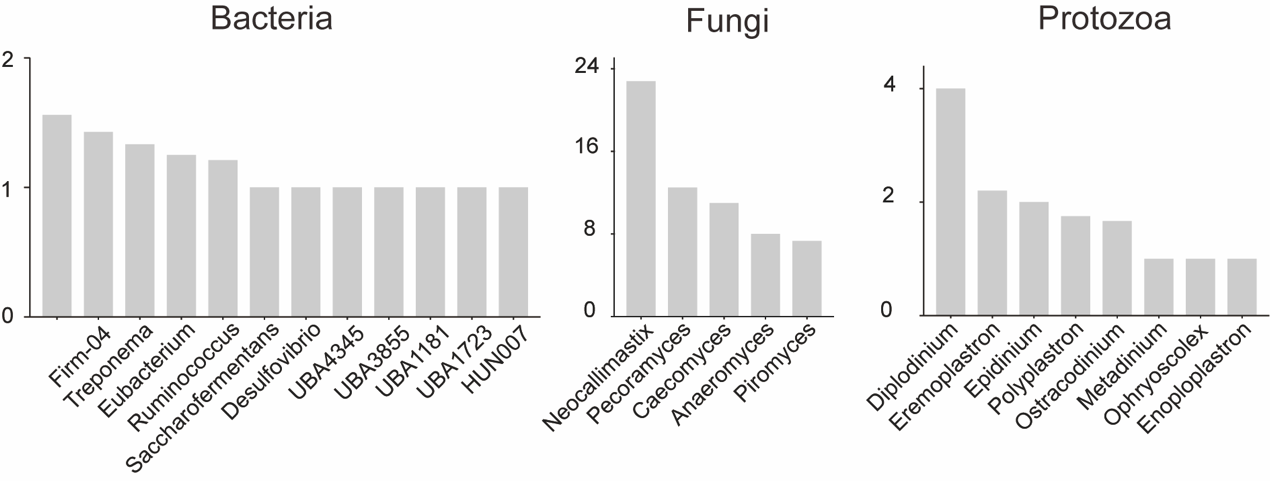


Figure S4. Analysis of mean ELP-encoding gene counts per genome at the genus level for rumen bacteria, fungi, and protozoa.


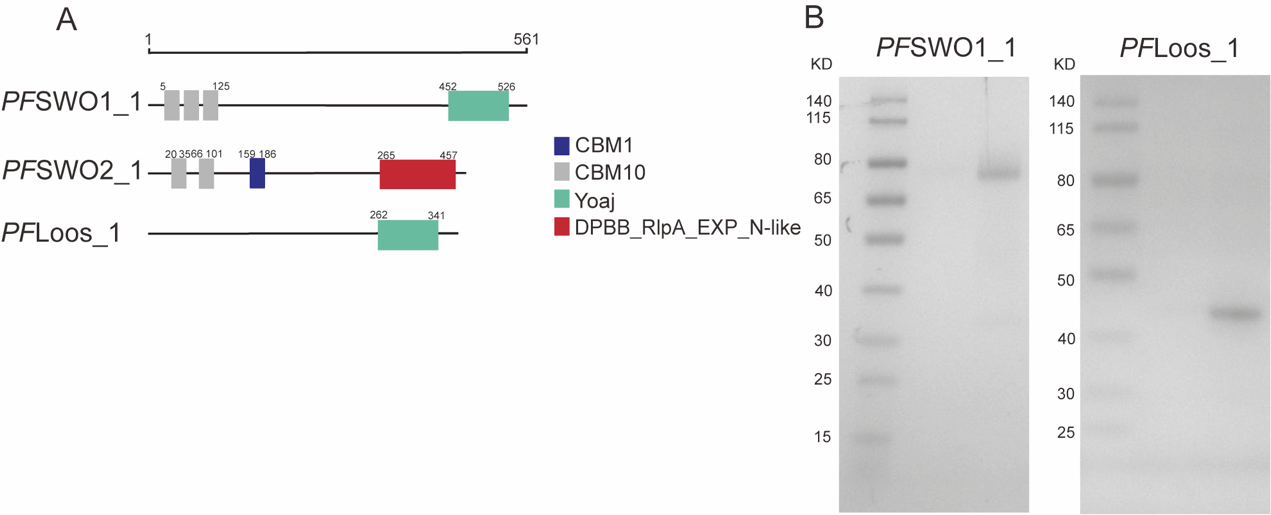


Figure S5. Expression and purification of selected expansin-like protein (ELP) from *Pecoramyces ruminantium* F1. (A) Domain architecture of representative highly expressed ELPs: CBM1 (blue), CBM10 (gray), Yoaj (green), and DPBB (red) domains. Numbers indicate amino acid positions at domain boundaries. (B) SDS-PAGE analysis of purified recombinant ELPs.
